# Supplementary material for: Genome-Wide Association Study Identified Novel Candidate Loci/Genes Affecting Lodging Resistance in Rice
Source: Genes (Basel). 2021 May 11;12(5):718. doi: 10.3390/genes12050718 (PMC8151605; doi:10.3390/genes12050718)
Supplement: Supplementary file 1 [file genes-12-00718-s001.zip › genes-1202549-supplementary.pdf]

Supplementary Figures and Tables

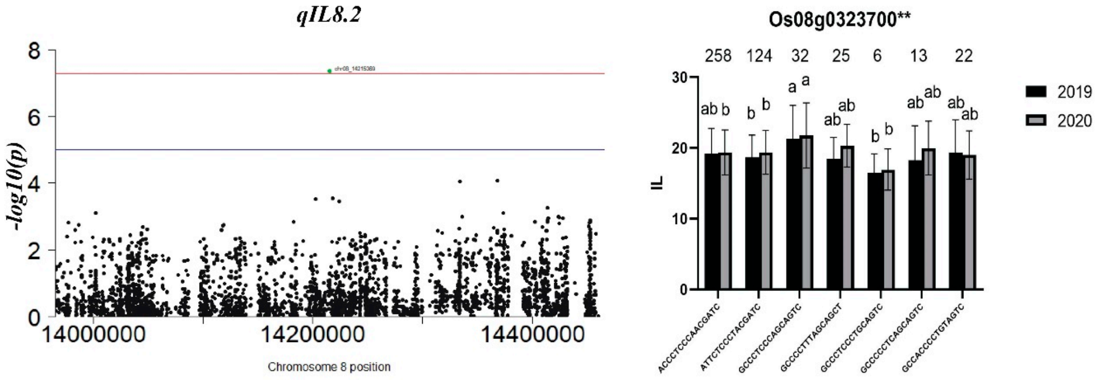

Figure S1. Gene-based association analysis of QTL(*qIL8.2*) loci and haplotypes analysis of targeted genes (*Os08g0323700*)**.** The ** suggested significance of ANOVA (for ≥ three haplotypes) or t-test (for two haplotypes) at p < 0.01. The letter on histogram (a, and b) indicated multiple comparisons result at the significant level 0.01. The value on the histogram was the number of individuals of each haplotype. Black and gray color indicated 2019 and 2020, respectively.

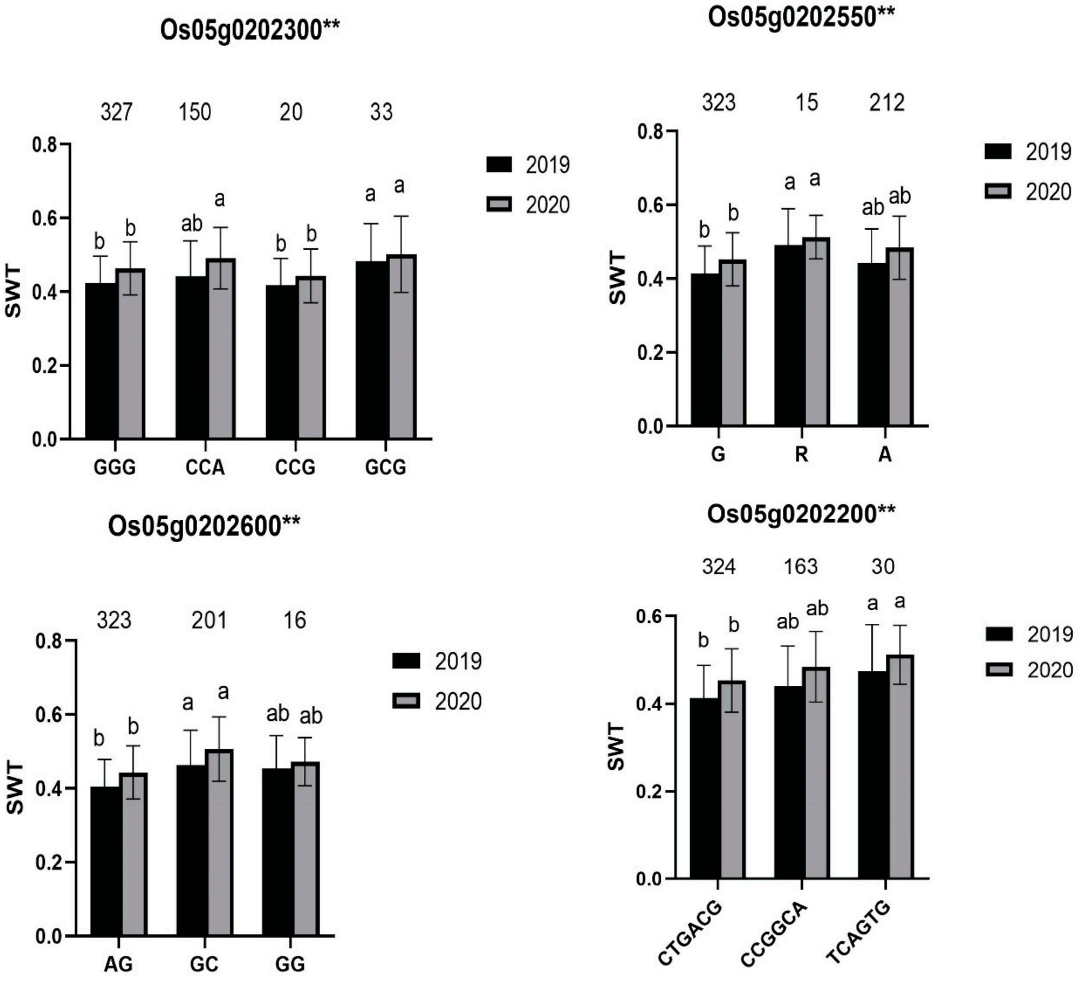

Figure S2. Haplotypes analysis of targeted genes of *qSWT5.2*. The ** suggested significance of ANOVA (for ≥ three haplotypes) or t-test (for two haplotypes) at p < 0.01. The letter on histogram (a, and b) indicated multiple comparisons result at the significant level 0.01. The value on the histogram was the number of individuals of each haplotype. Black and gray color indicated 2019 and 2020, respectively.

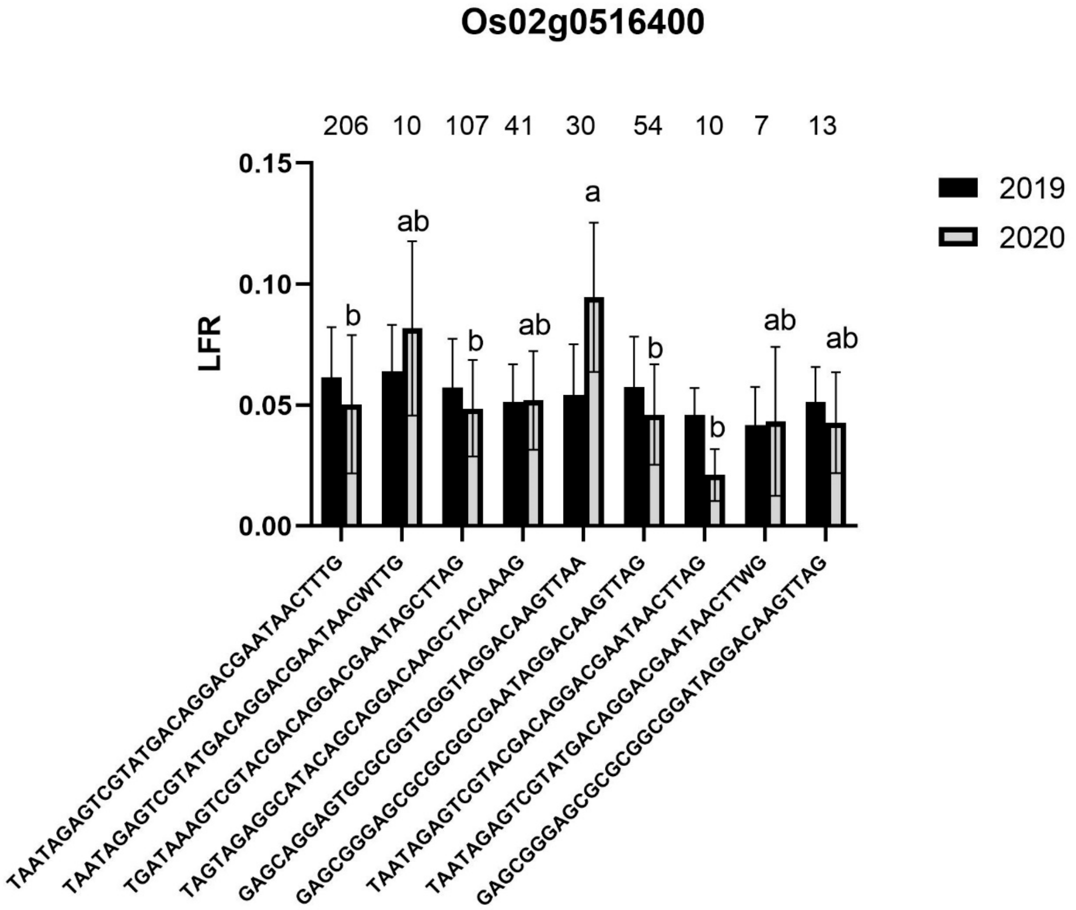

Figure S3. Haplotypes analysis of genes (*Os02g0516400*)**.** The ** suggested significance of ANOVA (for ≥ three haplotypes) or t-test (for two haplotypes) at $p < 0.01$. The letter on histogram (a, and b) indicated multiple comparisons result at the significant level 0.01. The value on the histogram was the number of individuals of each haplotype. Black and gray color indicated 2019 and 2020, respectively.

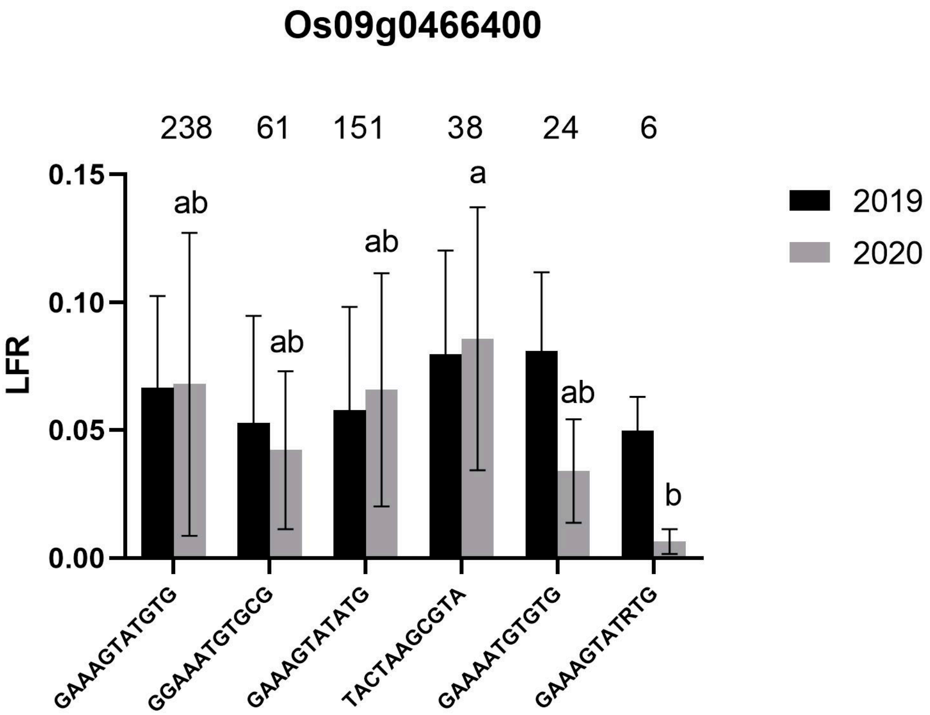

Figure S4. Haplotypes analysis of genes (*Os09g0466400*). The ** suggested significance of ANOVA (for ≥ three haplotypes) or t-test (for two haplotypes) at $p < 0.01$. The letter on histogram (a, and b) indicated multiple comparisons result at the significant level 0.01. The value on the histogram was the number of individuals of each haplotype. Black and gray color indicated 2019 and 2020, respectively.

Table S1. List of the 550 varieties used this study

Serial number	Variety name	Serial number	Variety name
Line 1	Chenghui448	Line 276	3210
Line 2	Budda	Line 277	CN1067
Line 3	Minghui63	Line 278	IR 10120-7-2-1-4
Line 4	Chorofa	Line 279	MBEIMBEIHUN
Line 5	D11	Line 280	MURGI BRINJ
Line 6	D15	Line 281	ZACATEPEC
Line 7	KR200	Line 282	71011
Line 8	829	Line 283	TAISEN GLUTINOUS YU 1157
Line 9	MERLE	Line 284	FEDEARROZ 50
Line 10	RR 272-17-829	Line 285	Kui630
Line 11	K 479-2-3	Line 286	Taizhong65/TaizhongHR539
Line 12	Zaoxian14	Line 287	PR106
Line 13	IRGA 959-1-2-2F-4-1-4A-6-CA-6X	Line 288	Padi Ladang Ase Polo Komek
Line 14	IR65600-27-1-2-2	Line 289	Chengnongshuijing
Line 15	Zhongjian100	Line 290	LUBUK LINGGAU
Line 16	E 2040	Line 291	Taizhongxianxuan2
Line 17	Guangluai4	Line 292	CH1157

Line 18	Zhongyouzao81	Line 293	Meihuanuo
Line 19	E ZI 96	Line 294	Muguanuo
Line 20	IRGA 370-38-1-1F-C4-2	Line 295	ARC 7425
Line 21	BHAINSA MUNDARIYA	Line 296	Xuanenchangtanqingzhan
Line 22	IR 2344-P1 PB-9-3-2B	Line 297	Baikehanhe
Line 23	Zhenshan97	Line 298	HSIEH-DAU
Line 24	ARROZVERMELHO	Line 299	SPR7284-57-5
Line 25	117	Line 300	AUS 449
Line 26	MONOLAYA	Line 301	Xianghui91269
Line 27	OM997	Line 302	R 762
Line 28	LALSAITA	Line 303	SHWEWARHNAN
Line 29	IRGA 659-1-2-2-2	Line 304	BAI MANG AI ZHONG
Line 30	DANAU LAUT TAWAR	Line 305	Jing87-304
Line 31	IR 57920-AC-25-2-B	Line 306	R8006(Zhonghui8006)
Line 32	WAS 173-B-B-6-2-2	Line 307	IR36
Line 33	ARAURE 1	Line 308	IR38（IR2070-423-2-5-6）
Line 34	AMISTAD 82	Line 309	IR64（IR18348-36-3-3）
Line 35	ICTA CRISPO 38	Line 310	IR6
Line 36	IR 75870-5-8-5-B-1	Line 311	IR72
Line 37	IR77298-14-1-2	Line 312	Minghui86
Line 38	Bala	Line 313	Kanghui63
Line 39	IR55419-04	Line 314	Luhui63
Line 40	Matatag2	Line 315	Ce49（Ce64-7-49）
Line 41	ARC 10100	Line 316	Ce64
Line 42	UNNAMED	Line 317	Duohui1
Line 43	Ajaya	Line 318	Mianhui734
Line 44	P59279	Line 319	Wanhui88
Line 45	Guang122	Line 320	R288
Line 46	IRAT144	Line 321	6078 (Yuhui6078）
Line 47	93072	Line 322	Shengtai1
Line 48	Maravilha	Line 323	Hua-2
Line 49	BG304	Line 324	Manghui
Line 50	YZX1	Line 325	Zhenhui084
Line 51	42686	Line 326	R287
Line 52	TKM 9	Line 327	Chenghui178
Line 53	CHANDINA	Line 328	Enhui69
Line 54	YA NONG ZAO 4	Line 329	R1128
Line 55	YSBR1	Line 330	II-32B
Line 56	BD007	Line 331	Jin23B
Line 57	E 2024	Line 332	WuxiangB
Line 58	GLADIO	Line 333	V20B
Line 59	Karnal Local	Line 334	BoB
Line 60	Y134	Line 335	MaxieB
Line 61	Amol3(Sana)	Line 336	CTB

Line 62	Bg 300	Line 337	898B
Line 63	BEGMI 135	Line 338	05CR89B
Line 64	YZX2	Line 339	EjinB
Line 65	IR64a	Line 340	Ezao18
Line 66	CALIFORNIA BELLE	Line 341	Yue4B
Line 67	WAS 206-B-B-2-2-1	Line 342	Runzhu537（Ezhong5）
Line 68	WAS 198-B-3-1-3	Line 343	Yuexiangzhan
Line 69	IR 56	Line 344	Xiangdao
Line 70	IR8192-200-3-3-1-1	Line 345	Molixiangzhan
Line 71	BA SHI ZAO	Line 346	Gaodanbaixiangdao
Line 72	Y075	Line 347	Jianzhen2
Line 73	NERICA-L-27	Line 348	BG304
Line 74	Molizhanxuan	Line 349	Bataixiangzhan
Line 75	Wanxian763	Line 350	Huhan3
Line 76	CT 6510-24-1-2	Line 351	Yangdao4
Line 77	SONA(IET 1991)	Line 352	Milyang83（Miyang83）
Line 78	Fengaizhan	Line 353	Wenshengnuo
Line 79	Yuexiangzhan	Line 354	Ewan13
Line 80	Hnankar	Line 355	Wen229
Line 81	IR68552-55-3-2	Line 356	Zaoyou143（ Xiangzao143）
Line 82	OM1706	Line 357	Yuanfengzao
Line 83	IRBB7	Line 358	Enhui58
Line 84	IR64-IL	Line 359	Guanghui380
Line 85	IR68897B	Line 360	Guanghui880
Line 86	PSBRC82	Line 361	158B
Line 87	NERICA-L-1	Line 362	814B （Jingchu814B）
Line 88	Huhan15	Line 363	Miyang83
Line 89	Giza14	Line 364	Gunong2923
Line 90	UPL RI-7	Line 365	Guojisuo1
Line 91	B 6144-MR-6-0-0	Line 366	Guooyou12
Line 92	GIZA 178	Line 367	#02428
Line 93	SAMBALA MALO	Line 368	Zhaiyeqing8
Line 94	WAS 199-B-1-2-1	Line 369	Forbiprotife
Line 95	IRI 339	Line 370	Ezao6
Line 96	IR 57514-PMI 5-B-1-2	Line 371	Ezao11
Line 97	Jiangxisimiao	Line 372	Xiangzaoxian21
Line 98	X21	Line 373	Zhenguiai
Line 99	C70	Line 374	ShijinB
Line 100	LX2007	Line 375	Taizhong1
Line 101	MAYBELLE	Line 376	Tetep （Tetepu）
Line 102	EX FOILAEIN(NAPUTO)	Line 377	Digu
Line 103	IR 21015-72-3-3-3-1	Line 378	C101LAC
Line 104	UPR 1201-1-20-1	Line 379	75-1-127
Line 105	RPW9-4(SS1)	Line 380	CBB23

Line 106	R644	Line 381	IRBB21
Line 107	Carijo	Line 382	Feng986
Line 108	IET1444	Line 383	Aizaizhan
Line 109	Ganwanxian37（926）	Line 384	Guongchangai3784
Line 110	Huangsizhan	Line 385	Qingfengai
Line 111	IR 2003-P7-7-4-2	Line 386	Jiangerai
Line 112	SANHUANGZHAN NO 2	Line 387	Qingerai
Line 113	IR 80310-12-B-1-3-B	Line 388	Jiduilun
Line 114	Bg90-2	Line 389	Kuoyedao
Line 115	Govind	Line 390	Yeqinglun
Line 116	NSIC RC9 (APO)	Line 391	Lucai
Line 117	Hua565	Line 392	Huanan15
Line 118	PT60	Line 393	Guangchangai4182
Line 119	KinandangPatong	Line 394	Huiyangai49
Line 120	BR11	Line 395	Zhaoyangzao18
Line 121	Huajingxian74	Line 396	Guichao2
Line 122	IR66897B	Line 397	Guangchang13
Line 123	IR58025B	Line 398	Fengqingai
Line 124	Qb_604	Line 399	Qingnongai
Line 125	Zhonghua1	Line 400	Fengaizhan1
Line 126	KCD1	Line 401	Huanghuazhan
Line 127	Nionoka	Line 402	Huangxinzhan
Line 128	CYPRESS	Line 403	28zhan
Line 129	Shwe Thwe Yin Hyv	Line 404	Fenghuazhan
Line 130	PSBRC88	Line 405	Qingliuai1
Line 131	R106	Line 406	Fengbaizhan
Line 132	X23	Line 407	Changsizhan
Line 133	C71	Line 408	Huasizhan
Line 134	IRBB62	Line 409	Yangdao2
Line 135	ZH5	Line 410	Teqing
Line 136	Jincouzhuxing	Line 411	Yangdao6
Line 137	NPT-100	Line 412	Sadu-cho
Line 138	EPEAL 102	Line 413	Shan-Huang Zhan
Line 139	WP65	Line 414	IR64
Line 140	PATISAIL	Line 415	N22
Line 141	2004	Line 416	Aijiaonante
Line 142	At354	Line 417	Guangluai4
Line 143	IR74	Line 418	Dianrui409B
Line 144	1088	Line 419	88B
Line 145	Hua564	Line 420	Xianghui91269
Line 146	E 2070	Line 421	Lucai
Line 147	Erjiunan1	Line 422	Chengduai3
Line 148	Lai nok kha	Line 423	Jiabala
Line 149	XIANG CHANG ZAO	Line 424	Taishannuo

Line 150	CHINA 1039 MUTANT(DWARF)	Line 425	Guichao2
Line 151	Aijiaonante	Line 426	Luke3
Line 152	LIU LI YOU	Line 427	Teqingxuanhui
Line 153	RONG DAO 4	Line 428	Huangsiguizhan
Line 154	LUO AI ZAO 3	Line 429	Xiangwanxian3
Line 155	M 6034-1	Line 430	Jinyou1
Line 156	KWANG-LU-AI 4	Line 431	Chengnongshuijing
Line 157	E ZI 32	Line 432	Momi
Line 158	Guangluai4	Line 433	Sanbaili
Line 159	JinnanteB	Line 434	Liusha1
Line 160	Sanbaili	Line 435	Dongtingwanxian
Line 161	HUO QING	Line 436	Yangdao2
Line 162	L 301B	Line 437	Liushizao
Line 163	SXC216	Line 438	Taizhongxianxuan2
Line 164	Yuenanzaodao	Line 439	Nante
Line 165	Chaoyang1B	Line 440	Heidu4
Line 166	Jinnante43B	Line 441	Jinnante43B
Line 167	Jiangnongzao1B	Line 442	80B
Line 168	AN FU ZHAN	Line 443	Aituogu151
Line 169	ZhuzhenB	Line 444	Menjiading2
Line 170	Zaoxian240	Line 445	Jiefangxian
Line 171	Baoxie-7B	Line 446	Baikehualuo
Line 172	LONG GE 33	Line 447	Liuyezhan
Line 173	Gzhenshan97B	Line 448	Zhenshan97
Line 174	KANGRI	Line 449	Minghui63
Line 175	Xiangzaoxiang7	Line 450	9311
Line 176	Aus/boro	Line 451	IRAT109
Line 177	Zaoshunonghu6	Line 452	Huajingxian74
Line 178	DECHANGBYEO	Line 453	Zihui100
Line 179	Lucai	Line 454	Varylava
Line 180	Qiuqianbai	Line 455	D62B
Line 181	AIJIAONANTE	Line 456	G46B
Line 182	IR 9747 SEL	Line 457	IR58025B
Line 183	HONG YANG ZAO 3	Line 458	Zhong9B
Line 184	Dongtingwanxian	Line 459	Xiang5
Line 185	Shufeng101	Line 460	Menjiagao2
Line 186	XiangaiB	Line 461	Exiang1
Line 187	ZHAO DAN AI	Line 462	H198-yeza
Line 188	Zhenfu8	Line 463	Huangxiuzhan
Line 189	Chenwan3	Line 464	Shuhui527
Line 190	Xiangwanxian1	Line 465	Shuhui288
Line 191	KAMULI	Line 466	Shuhui707
Line 192	CN44-40-7	Line 467	Longhui11

Line 193	SI WAN 14	Line 468	Nanhui511
Line 194	CR579-1-3	Line 469	Xianghui529
Line 195	KABERI	Line 470	Jinhui275
Line 196	Sililanka1	Line 471	Shanhui287
Line 197	LIANG GUANG	Line 472	Shanhui8281
Line 198	SHUANG BAI AI 2	Line 473	205R
Line 199	SUNAR	Line 474	781R
Line 200	W 398	Line 475	3301R
Line 201	Nante	Line 476	IR24
Line 202	DA GU AI 7	Line 477	IR1544
Line 203	JIANG ER ZAO	Line 478	Gui630
Line 204	PJ110	Line 479	Nanhui511
Line 205	GUANG XUAN LIU HAO	Line 480	Shuhui202
Line 206	ZUIHOU	Line 481	Chenghui727
Line 207	IR 77298-14-1-2-10	Line 482	Jinhui10
Line 208	Malaihong	Line 483	Jinhui18
Line 209	Dianrui409B	Line 484	Jinhui16
Line 210	531	Line 485	Jinhui35
Line 211	FACAGRO 406	Line 486	Wanhui88
Line 212	QUN XUAN ZAO	Line 487	Wanhui66
Line 213	IRAT 10	Line 488	Fuhui802
Line 214	Guangluai15-1	Line 489	Huhui602
Line 215	Mamagu	Line 490	Chenghui881
Line 216	ARC 11777	Line 491	Yihui3551
Line 217	FAN WU	Line 492	Fuhui838
Line 218	E ZI 110	Line 493	Minhui3301
Line 219	Xiaohonggu	Line 494	Yihui72
Line 220	Dangyu5	Line 495	Gui553
Line 221	78 XUAN WU	Line 496	Chenghui3203
Line 222	KUNJUKUNJU	Line 497	Ehui108
Line 223	PERUBAK LUEY	Line 498	Huarun2
Line 224	RP20-12	Line 499	Ganghui988
Line 225	GZ 1368-5-4	Line 500	Wusansimiao
Line 226	Aituogu151	Line 501	E1573
Line 227	E ZI 100	Line 502	R-Zh
Line 228	CUN GU NUO	Line 503	S112xuan
Line 229	ITA 117	Line 504	Ewan17B
Line 230	NCS349	Line 505	EX MARABA-GURUKU
Line 231	QING TAI AI	Line 506	Haogelao
Line 232	PL 3165	Line 507	IR 28
Line 233	Xugunuo	Line 508	C418
Line 234	IR 19058-107-1	Line 509	KURULU WEE(WHITE)
Line 235	IR 3839-1	Line 510	FIDJI

Line 236	RATNAGIRI 45-2
Line 237	PORONG
Line 238	TAICHUNG SEN-YU 214
Line 239	B 737G-KN-23-1
Line 240	IR 73571-3B-11-3-K2
Line 241	LUAN DAO
Line 242	Youzhan
Line 243	Chengduai3
Line 244	88B
Line 245	Nanjing11
Line 246	Zhenxian232
Line 247	Liusha1
Line 248	BuleidaA-75
Line 249	Wanhong1
Line 250	JIN HUA 258
Line 251	KA GIL
Line 252	AR 133
Line 253	HSINCHU AI CHIO CHIENG
Line 254	TAIPEI 167
Line 255	IR 77186-122-2-2-3
Line 256	Jiabala
Line 257	Teqingxuanhui
Line 258	JWR 221
Line 259	Huangsiguizhan
Line 260	Gongju73
Line 261	Luke3
Line 262	BOUAKE 189
Line 263	PDR 34-2-1-2
Line 264	NAN-ERH-AI 5
Line 265	SI CHAO 1
Line 266	TE SAN AI 2
Line 267	ALTAMIRA 9
Line 268	C 166-135
Line 269	IR 63295-AC 209-7
Line 270	IR 5657-33-2
Line 271	TOS 9795
Line 272	K 24
Line 273	13946(GUANG-QIU 15)
Line 274	RP 1570-44-1
Line 275	Guichao2
Line 511	TAK SUFAID
Line 512	SLAVA
Line 513	SAHEL 108
Line 514	BATHURI
Line 515	DALSUNG 41
Line 516	DANGAR
Line 517	B 6136-3-TB-0-1-5
Line 518	Zhongchao123
Line 519	Gang46B
Line 520	CO 39
Line 521	GRITNA
Line 522	JAGLI BORO
Line 523	Gaozi
Line 524	BINUHANGIN
Line 525	Yanghei3
Line 526	Zixiang3
Line 527	Hongxiang2
Line 528	Lvxiang3
Line 529	Huangxiang2
Line 530	Hunanningxiangyuanzinuo
Line 531	Hunanningxiangchangzinuo
Line 532	Guangximomi
Line 533	Niboerhuangdao
Line 534	DYheidao
Line 535	Jiangxishangraozixiangmi
Line 536	Guangxishanglinhongxiangmi
Line 537	Fujianyouxiheiguomi
Line 538	Fujianyouxihongguomi
Line 539	Yuenanhongmi
Line 540	Luhui17
Line 541	15HP58
Line 542	Luohui69
Line 543	Zaohui43
Line 544	Zaohui49
Line 545	R9519
Line 546	R5431
Line 547	900
Line 548	Feng9
Line 549	1128
Line 550	9348

Grey filling materials come from 3K RGP, in total of 327 varieties .

Table S2. QTL identified for traits in 2019 and 2020 in Wuhan, China.

QTL	year	CHROM	POS	REF	ALT	Effect	SE	p
qLFR2.1	2019	2	4243076	G	A	0.06	0.011	1.17E-07
	2020	2	4461960	G	T	0.05	0.010	4.24E-08
qLFR2.2	2019	2	7856970	C	T	-0.02	0.004	1.85E-07
qLFR2.3	2019	2	18545189	A	G	0.03	0.005	1.82E-07
qLFR3.1	2020	3	7298950	G	A	0.08	0.012	1.48E-11
qLFR3.2	2019	3	8085748	T	C	0.05	0.009	1.10E-08
qLFR3.3	2019	3	13390842	C	A	0.03	0.005	1.59E-08
qLFR3.4	2019	3	19705332	A	G	0.03	0.005	1.69E-08
qLFR3.5	2019	3	36384565	C	A	0.03	0.005	2.23E-07
qLFR4.1	2020	4	731040	G	A	0.06	0.011	1.66E-07
qLFR4.2	2019	4	4933337	A	G	0.02	0.004	1.24E-07
qLFR4.3	2019	4	7087567	G	A	0.02	0.004	6.56E-08
qLFR4.4	2020	4	10939548	T	C	0.05	0.009	1.01E-08
qLFR4.5	2019	4	25161106	C	T	0.03	0.006	2.62E-08
qLFR5.1	2019	5	10941409	G	A	0.03	0.006	4.10E-08
qLFR5.2	2019	5	15919313	C	T	0.04	0.006	2.59E-08
qLFR5.3	2020	5	20540653	A	G	0.06	0.011	1.42E-08
	2019	5	20540653	A	G	0.05	0.009	4.64E-08
qLFR5.4	2019	5	21734179	G	A	0.03	0.006	7.10E-10
qLFR5.5	2019	5	23889281	T	C	-0.02	0.004	1.80E-07
qLFR6.1	2020	6	3467131	G	A	0.05	0.009	1.08E-09
qLFR6.2	2019	6	15151322	C	T	0.03	0.006	2.31E-07
qLFR6.3	2020	6	18858265	A	C	0.03	0.007	1.86E-07
qLFR7.1	2019	7	7580625	G	A	0.03	0.006	2.34E-07
qLFR7.2	2019	7	17459043	C	A	0.03	0.006	1.71E-07
qLFR7.3	2020	7	19788362	C	A	0.03	0.007	5.20E-08
qLFR8.1	2019	8	17303967	T	C	0.02	0.005	2.16E-07
qLFR8.2	2019	8	27750738	G	A	0.04	0.007	1.33E-10
qLFR8.3	2020	8	28136250	G	A	0.07	0.012	7.10E-13
qLFR9.1	2019	9	17764668	C	A	0.04	0.006	3.06E-08
qLFR9.2	2019	9	18757001	T	G	0.02	0.004	2.23E-07
qLFR10	2020	10	11069031	C	A	-0.05	0.009	2.48E-08
qLFR11.1	2019	11	2616479	A	G	0.03	0.005	6.98E-10
qLFR11.2	2019	11	3675601	A	T	0.02	0.005	1.68E-07
qLFR12	2019	12	11842272	G	A	0.03	0.008	5.33E-11
qSID1	2020	1	16559456	T	C	0.38	0.068	5.48E-08
qSID2	2019	2	4246890	C	T	-0.23	0.043	1.38E-09
qSID3	2019	3	14025928	A	G	-0.21	0.040	6.83E-09
qSID5.1	2019	5	9683471	T	C	0.20	0.047	1.45E-07
qSID5.2	2019	5	10353586	G	A	0.17	0.035	7.95E-08
qSID6.1	2020	6	14785782	T	A	0.42	0.079	1.80E-07
qSID6.2	2019	6	23317069	C	T	0.12	0.029	4.19E-08
qSID6.3	2020	6	28754862	G	A	0.34	0.063	1.34E-07
qSID7	2020	7	9226778	C	A	0.37	0.069	1.13E-07
qSID8.1	2020	8	8673481	G	A	0.28	0.050	4.97E-08
	2019	8	8722341	C	T	0.35	0.053	1.32E-10
qSID8.2	2019	8	14658311	G	A	-0.30	0.060	2.43E-08
qSID8.3	2020	8	23122614	A	C	0.40	0.076	1.87E-07
qSID9.1	2019	9	17873714	T	C	0.25	0.049	6.83E-09
qSID9.2	2019	9	21753476	A	G	0.21	0.032	1.54E-12
qSID10.1	2019	10	13419306	T	A	0.20	0.042	6.43E-08
qSID10.2	2019	10	14781430	C	T	0.22	0.042	9.49E-09
qSID12.1	2019	12	3325937	T	C	-0.25	0.050	2.89E-09
qIL1.1	2019	1	5920879	C	T	1.85	0.341	8.34E-08
qIL1.2	2020	1	32933806	G	T	1.65	0.299	6.10E-08
	2019	1	32934166	T	C	1.61	0.300	1.39E-07

qIL4.1	2020	4	8874974	A	T	1.94	0.366	1.95E-07
qIL4.2	2020	4	13394666	C	T	1.63	0.311	2.30E-07
qIL6	2019	6	10410709	A	G	1.75	0.329	1.62E-07
qIL8.1	2019	8	8396436	C	T	1.50	0.283	2.32E-07
	2020	8	8717396	C	T	1.75	0.334	1.84E-07
qIL8.2	2020	8	14215369	G	A	1.70	0.304	4.22E-08
qIL8.3	2019	8	19789417	T	C	1.11	0.209	1.72E-07
qIL8.4	2019	8	20788042	C	T	-3.43	0.648	1.82E-07
qIL9	2019	9	9025294	C	T	-2.42	0.422	1.75E-08
qSPAD5	2020	5	11676591	C	T	-2.85	0.514	4.72E-08
qSPAD6	2020	6	6640251	A	G	1.65	0.312	1.96E-07
qSPAD7	2019	7	5119605	G	A	-1.12	0.212	1.92E-07
qSPAD10	2019	10	121721	A	G	-2.02	0.363	4.61E-08
qSPAD11.1	2020	11	27695597	C	A	-1.54	0.284	1.00E-07
qSPAD11.2	2020	11	28410048	A	G	-1.47	0.277	1.89E-07
qSWT1	2019	1	7415781	C	T	-0.04	0.009	1.73E-07
qSWT3	2019	3	8841153	T	G	0.03	0.006	9.89E-08
qSWT4.1	2019	4	5070468	A	G	-0.02	0.004	1.20E-08
qSWT4.2	2019	4	6029011	C	T	-0.04	0.008	2.96E-08
qSWT4.3	2019	4	29983581	T	A	-0.03	0.008	1.59E-07
	2020	4	30459506	A	G	-0.06	0.010	9.45E-08
qSWT4.4	2019	4	31840365	G	A	-0.03	0.005	2.56E-09
qSWT5.1	2020	5	1755834	T	C	0.04	0.007	2.56E-08
qSWT5.2	2020	5	6243695	C	T	0.02	0.004	1.27E-07
	2019	5	6245355	C	T	0.01	0.003	1.97E-08
qSWT5.3	2020	5	8202592	C	G	-0.05	0.009	1.09E-07
qSWT6	2019	6	26041721	T	C	0.04	0.008	1.09E-08
qSWT7.1	2019	7	16854258	G	A	0.04	0.007	1.92E-09
qSWT7.2	2019	7	25723398	C	A	-0.03	0.006	6.26E-09
qSWT7.3	2019	7	28576217	T	C	-0.07	0.013	2.06E-07
	2020	7	28576224	T	C	-0.07	0.013	2.06E-07
qSWT8	2019	8	7207333	T	C	-0.02	0.004	1.06E-09
qSWT9	2019	9	14136274	C	T	-0.01	0.003	9.57E-08
qSWT12	2019	12	8606248	T	C	0.05	0.010	1.69E-07
qFLW2	2020	2	18692065	T	C	0.19	0.034	3.13E-08
qFLW3	2020	5	20496180	C	T	0.17	0.031	1.35E-07
qFLW8	2020	8	22563133	T	C	0.22	0.041	8.67E-08
	2019	8	22849319	C	T	0.21	0.038	4.24E-08
qFLA2.1	2020	2	17841259	C	T	2.90	0.485	4.95E-09
qFLA2.2	2020	2	26744192	A	C	1.70	0.299	2.53E-08
qFLA5.1	2020	5	4747434	G	A	2.17	0.387	3.50E-08
qFLA5.2	2020	5	17413706	T	C	2.16	0.362	4.92E-09

Table S3. Candidate genes for each QTL.

Traits	QTL	Candidate genes
IL	*qIL1.1*	*Os01g0208600*
	qIL8.1	*Os08g0243100, Os08g0244500*
	qIL8.2	*Os08g0323700*
SWT	*qSWT5.2*	*Os05g0200160, Os05g0200340, Os05g0200400, Os05g0200500, Os05g0201300, Os05g0202200, Os05g0202300, Os05g0202550 ,Os05g0202600*
SID	*qSID8.1*	*Os08g0243100*, *Os08g0243500*
LFR	*qLFR2.3*	*Os02g0516400*
	qLFR9.1	*Os09g0471000, Os09g0471100 ,Os09g0471200*
SPAD	*qSPAD7*	*Os07g0192000*
	qSPAD11.1	*Os11g0682600*
